# Slow CCL2-dependent translocation of biopersistent particles from muscle to brain

**DOI:** 10.1186/1741-7015-11-99

**Published:** 2013-04-04

**Authors:** Zakir Khan, Christophe Combadière, François-Jérôme Authier, Valérie Itier, François Lux, Christopher Exley, Meriem Mahrouf-Yorgov, Xavier Decrouy, Philippe Moretto, Olivier Tillement, Romain K Gherardi, Josette Cadusseau

**Affiliations:** 1 Inserm, U955, 8 rue du Général Sarrail, Créteil, 94010, France; 2 Université Paris Est, Faculté de Médecine, 8 rue du Général Sarrail, Créteil, 94010, France; 3 Inserm, UMR-S 945, 91 Boulevard de l’Hôpital, Paris, 75013, France; 4 Université Pierre et Marie Curie, Faculté de Médecine, 11 Boulevard de l’Hôpital, Paris, 75013, France; 5 AP-HP, Groupe Hospitalier Pitié-Salpétrière, Service d’Immunologie, 11 Boulevard de l’Hôpital, Paris, 75013, France; 6 AP-HP, Hôpital H. Mondor - A. Chenevier, Service d’Histologie, Centre de Référence Neuromusculaire GNMH, 51 Avenue du Maréchal de Lattre de Tassigny, Créteil, 94000, France; 7 CNRS UMR 5620, Laboratoire de Physico-Chimie des Matériaux Luminescents, 2 rue Victor Grignard, Villeurbanne, 69622, France; 8 Université Claude Bernard Lyon 1, 2 rue Victor Grignard, Villeurbanne, 69622, France; 9 The Birchall Centre, Lennard-Jones Laboratories, Keele University, Staffordshire, ST5 5BG, UK; 10 CNRS UMR 5797, Centre d'Etudes Nucléaires de Bordeaux Gradignan, Allée du haut Vignaud, Gradignan, 33175, France; 11 Faculté des Sciences et Technologie, UPEC, 61 Avenue du Général de Gaulle, Créteil, France; 12 IMRB Team 10, Faculté de Médecine, 8 rue du Général Sarrail, Créteil, F-94010, France

**Keywords:** Alum, Vaccine adverse effect, Vaccine adjuvant, Nanomaterial biodistribution, Nanomaterial neurodelivery, Macrophages, Macrophagic myofasciitis, CCL-2, Single nucleotide polymorphisms (SNPs)

## Abstract

**Background:**

Long-term biodistribution of nanomaterials used in medicine is largely unknown. This is the case for alum, the most widely used vaccine adjuvant, which is a nanocrystalline compound spontaneously forming micron/submicron-sized agglomerates. Although generally well tolerated, alum is occasionally detected within monocyte-lineage cells long after immunization in presumably susceptible individuals with systemic/neurologic manifestations or autoimmune (inflammatory) syndrome induced by adjuvants (ASIA).

**Methods:**

On the grounds of preliminary investigations in 252 patients with alum-associated ASIA showing both a selective increase of circulating CCL2, the major monocyte chemoattractant, and a variation in the *CCL2* gene, we designed mouse experiments to assess biodistribution of vaccine-derived aluminum and of alum-particle fluorescent surrogates injected in muscle. Aluminum was detected in tissues by Morin stain and particle induced X-ray emission) (PIXE) Both 500 nm fluorescent latex beads and vaccine alum agglomerates-sized nanohybrids (Al-Rho) were used.

**Results:**

Intramuscular injection of alum-containing vaccine was associated with the appearance of aluminum deposits in distant organs, such as spleen and brain where they were still detected one year after injection. Both fluorescent materials injected into muscle translocated to draining lymph nodes (DLNs) and thereafter were detected associated with phagocytes in blood and spleen. Particles linearly accumulated in the brain up to the six-month endpoint; they were first found in perivascular CD11b^+^ cells and then in microglia and other neural cells. DLN ablation dramatically reduced the biodistribution. Cerebral translocation was not observed after direct intravenous injection, but significantly increased in mice with chronically altered blood-brain-barrier. Loss/gain-of-function experiments consistently implicated CCL2 in systemic diffusion of Al-Rho particles captured by monocyte-lineage cells and in their subsequent neurodelivery. Stereotactic particle injection pointed out brain retention as a factor of progressive particle accumulation.

**Conclusion:**

Nanomaterials can be transported by monocyte-lineage cells to DLNs, blood and spleen, and, similarly to HIV, may use CCL2-dependent mechanisms to penetrate the brain. This occurs at a very low rate in normal conditions explaining good overall tolerance of alum despite its strong neurotoxic potential. However, continuously escalating doses of this poorly biodegradable adjuvant in the population may become insidiously unsafe, especially in the case of overimmunization or immature/altered blood brain barrier or high constitutive CCL-2 production.

## Background

Nanomaterials have various innovative medical applications including drug and gene delivery, imaging contrast fluids, topical antimicrobials, surgery tools and vaccines [1]. Due to the growing number of novel compounds and formulations, data on their specific biodistribution, persistence and toxicity are generally lacking [1], and clarification regarding how the body handles small particles, especially those which interact with immune cells [2], is urgently needed. Once defined, these basic mechanisms which govern host-particle interactions should be integrated with specific properties of nanomaterials (size, shape, surface, and solubility) to enable predictions of their beneficial or adverse effects.

The use of nanomaterials in humans is not as contemporary as is recently portrayed. For decades, alum, a nanocrystalline compound formed of aluminum oxyhydroxide, has been the most commonly used adjuvant in vaccines. The mechanism by which it stimulates the immune response is incompletely understood [3]. While alum is generally well tolerated, it is occasionally reported as the cause of disabling health problems in individuals with ill-defined susceptibility factors [4-6]. Clinical manifestations attributed to alum are paradigmatic of the so-called autoimmune/inflammatory syndrome induced by adjuvants (ASIA), a syndrome also observed in patients exposed to silicone gel [7]. They include delayed onset of diffuse myalgia [4], chronic fatigue [8] and stereotyped cognitive dysfunction [9]. The persistence of alum-loaded macrophages is typically detected at sites of previous injections (up to >12 years later), resulting in a specific granuloma called macrophagic myofasciitis or MMF [4]. Although the biopersistence of adjuvants is *a priori* undesirable, the exact significance of this remains the subject of some debate since the biodistribution of slowly biodegradable particles following injection into muscle is currently unknown.

There appears to be a fine balance between the efficacy of alum adjuvant and its potential toxicity, and there is good evidence that these may be one and the same effect [3]. Both the efficacy and the potential toxicity of alum will be influenced by whether the bioactive nanomaterial remains localized at injection points or rather scatters and accumulates in distant organs and tissues. A reference study based on isotopic ^26^Al showed poor (6%) ^26^Al clearance in the urine at the day 28 (d28) endpoint after i.m. injection of isotopic alum to rabbits, and detected ^26^Al, in an unknown form, in lymph nodes, spleen, liver, and brain [10]. Aluminum oxyhydroxide is composed of micron/submicron-sized aggregates of nano-sized (about 13 nm) particles and these aggregates were initially believed to remain extracellular until their complete solubilization in interstitial fluids [10]. We now know that quite the reverse is the case and that antigen presenting cells (APCs) avidly take up alum particles [11], and, in so doing, become long-lived cells [12] and impede alum solubilization [4,13,14]. Inflammatory monocytes (MOs) are attracted into muscle by danger signals through a monocyte chemoattractant protein-1 (MCP-1)/chemokine (C-C motif) ligand 2 (CCL2) driven-mechanism, becoming macrophages (MPs) and MO-derived dendritic cells (DCs), before migrating to the draining lymph nodes (DLNs) [15]. One function of migratory DCs is to transfer antigenic material to a large network of distant resident APCs [16]. Moreover, injections of alum alone induce significant changes linked to activation of the innate immune system in distant organs [17,18]. Therefore, we examined whether nanomaterials injected into muscle could translocate to distant organs as part of a general mechanism linked to phagocytosis and CCL2/MCP-1 signaling.

## Methods

### Mice models

All animal experiments were conducted in accordance with the European guidelines for animal care. To facilitate mechanistic investigation of particle biodistribution, mice of the B57/B6 genetic background, that are used to generate genetically-manipulated models, were preferred to more toxic-sensitive mouse strains. Male eight- to ten-week-old C57BL/6, *mdx* (with leaky blood brain barrier (BBB)), *CX3CR1*^GFP/+^ (with GFP reporter gene insertion allowing visualization of microglia), and *CCL2*^−/−^ mice were used (Jackson, West Grove, PA, USA). Mice were protected from Al-containing materials, fed with manufactured animal food and water *ad libitum*, and exposed to 12:12 light/dark cycles. Experiments using fluorescent particles were extremely labor intensive and expensive to perform. All of them were done in triplicate. Homogeneity of results made it unnecessary to use more than three mice per point.

### Alum administration

The dose of alum-containing vaccine administered to mice was calibrated to mimic the mean number of doses received by MMF patients. One dose of commercially available anti-hepatitis B vaccine contains 0.5 mg Al according to the product data sheet. Based on an average of human body weight of 60 kg (most patients being women), the amount received for each immunization is 8.33 μg/kg. The allometric conversion from human to mouse (FDA Guidance 5541) gives a final amount of approximately 100 μg/kg. A dose of 36 μL vaccine, which corresponds to 18 μg Al, was injected to mimic the cumulative effect induced by 5.2 human doses to 35 g mice (the mean weight at the d180 midtime of brain analysis). This dose represents an equivalent 6.8 human doses in the youngest animal (27 g body weight, 11 weeks of age at sacrifice) and 4.3 in the oldest one (42 g at 62 weeks).

### Furnace atomic absorption spectrometry

Al concentrations were determined in whole tibialis anterior (TA) muscles and brains dried at 37°C and digested with concentrated HNO_3_ (14 mol/L). Digests were allowed to cool before dilution to 10% HNO_3_ with ultra-pure water. The total aluminum in each digest was measured by transversely heated graphite atomizer graphite furnace atomic absorption spectrometry (TH GFAAS) and results were expressed as Al mg/g tissue dry weight.

### PIXE

As in normal conditions Al may be detected with marked interindividual variations in tissues, *de novo* incorporation of aluminum in too low doses does not cause easily detectable changes when global conventional approaches are used [10]. Here we used particle induced X-ray emission (PIXE), a procedure analyzing radiation emitted from the interaction of a proton beam with the matter [19], to detect areas enclosing small Al spots. Sections (20 μm-thick) carefully protected from environmental Al were mounted on fresh formvar films, kept in the cryostat for 6 hours and stored under Al-free silica gel. Mineral and metal ions were detected using the nuclear microprobe of the Centre d’Etudes Nucléaires de Bordeaux-Gradignan. A 1 MeV proton beam focused down to a 2 μm spot was randomly scanned over multiple 500 × 500 μm fields of tissue sections. In the case of an Al signal, a re-test of 100 × 100 μm areas of interest was performed. PIXE and Rutherford backscattering spectrometry analyses were employed simultaneously and quantitative results were computed, as previously described [19]. Al spots were considered eligible on three criteria: a size of more than 3 pixels (that is, above the background noise), a depot not colocalized with Si, and a depot surrounded by a rounded halo of decreased intensity (both characteristics limiting confusion with contamination by external dust overcoming the protection procedures).

### Synthesis of Al-Rho particles

Gadolinium oxide nanohybrids with Al(OH)_3_ coating were obtained in three steps: (i) gadolinium oxide nanoparticles were first synthesized; (ii) polysiloxane shell growth was then induced by hydrolysis-condensation of convenient silane precursors in the presence of the nanoparticles; and (iii) the nanohybrids were coated by the addition of aluminum nitrate and soda in stoichiometric conditions.

#### Chemicals

Gadolinium chloride hexahydrate ((GdCl_3_, 6H_2_O]) 99.99%), sodium hydroxide (NaOH, 99.99%), tetraethyl orthosilicate (Si(OC_2_H_5_)_4_, TEOS, 98%), (3-aminopropyl) triethoxysilane (H_2_N(CH_2_)_3_-Si(OC_2_H_5_)_3_, APTES, 99%), triethylamine (TEA, 99.5%), rhodamine B isothiocyanate (RBITC), aluminium nitrate nonahydrate (Al(NO_3_)_3_.9H_2_O, ACS reagent ≥ 98%) and dimethyl sulfoxide (DMSO, 99.5%) were purchased from Sigma-Aldrich (St Louis, MO, USA). Diethylene glycol (DEG, 99%) was purchased from SDS Carlo Erba, Val de Reuil (France).

#### Preparation of gadolinium oxide core

A first solution was prepared by dissolving GdCl_3_, 6H_2_O (0.56 g) in 50 mL DEG at room temperature. A second solution was prepared by adding a NaOH solution (0.49 mL, 10 M) in 50 mL DEG. The second solution was progressively added to the first one, at room temperature, for 15 hours. A transparent colloid of gadolinium oxide nanoparticles in DEG was obtained.

#### Encapsulation of Gd_2_O_3_ cores by polysiloxane shell

A total of 105 μL of APTES and 67 μL of TEOS was added to 100 mL of the gadolinium oxide nanoparticle solution under stirring at 40°C. A total of 5 μL of APTES was previously coupled to 1 mg RBITC in DMSO (1 mL) used as solvent and then added to the colloidal solution. After 1 hour, 1,913 μL of a DEG solution (0.1 M of TEA, 10 M of water) was added. The whole coating procedure was repeated three more times (with no more addition of RBITC), every 24 hours. The final mixture was stirred for 48 hours at 40°C. The obtained solution could be stored at room temperature for weeks without alteration.

#### Coating of fluorescent nanohybrids with a Al(OH)_3_ shell

A total of 2.5 mL of the colloidal solution was diluted by 2 to obtain a 5 mL solution in DEG. A total of 75 mg of aluminum nitrate nonahydrate was dissolved in 10 mL of water before addition to the colloidal solution. The resulting mixture was stirred for 5 minutes and 4 mL of a soda solution (0.2 M) was added before stirring for 1 hour.

#### Purification

Purification of Al-Rho was performed by tangential filtration through Vivaspin filtration membranes (MWCO = 10 kDa) purchased from Sartorius Stedim Biotech (Aubagne, France). The colloidal solution was introduced into 20 mL Vivaspin tubes and centrifuged at 4,100 rpm. This step was repeated several times, by filling the tubes with water and centrifuging again, until the desired purification rate was reached (≥100). The purified colloidal solution was freeze dried for storage in five pillboxes, using a Christ Alpha 1–2 lyophilisator. The compound contained 4 μg Al per μL of Al-Rho suspension. Control transmission electron microscopy showed non-fibrous particles about 10 nm in size, typical of aluminum hydroxyde (traditional precipated alum). Similarly to vaccine alum, they formed agglomerates of submicronic/micronic size. The immunological properties of such traditional alum-protein precipitates are quite similar to those of the reference adjuvant approved by the FDA (Al oxyhydroxyde: Alhydrogel®, Invivogen, Toulouse France) and differ from other formulations not licensed for human use (18).

### Peripheral injections of fluorescent nanomaterials

Two types of fluorescent nanomaterials were used: exploratory polychromatic fluorescent latex beads (FLBs) (500 nm fluorospheres, Polysciences, Warrington, PA, USA) and confirmatory Al-Rho nanohybrids constructed with a rhodamine containing core and an Al(OH)_3_ shell. FLBs were used first because they offer several characteristics that facilitate their detection in tissues, including strong fluorescence, spheric appearance and homogeneous size. This allowed us to get a clear picture of what was happening in terms of biodistribution of these avidly phagocytosed particles. Al Rho particles were less fluorescent and more heterogeneous in shape and size than FLBs but better represented alum adjuvant surrogates. Almost all biodistribution experiments performed with FLBs in wild type mice were also done with Al-Rho. In contrast, FLBs and Al Rho were differentially used in mutated/genetically-modified mice: FLBs were preferred to study particle biodistribution in *mdx* mice with BBB alterations and when the GFP marker was used (that is, *CX3CR1*^GFP/+^ mice with fluorescent microglia, GFP + BMT studies); Al-Rho particles were preferred in gain/loss of CCL2/MCP-1 function studies designed on the basis of preliminary results on the CCL2 status of alum-intolerant humans.

FLB suspension diluted at 1:1 in PBS contained 1.8 × 10^11^ particles per mL. A total volume of 40 μL (20 μL in each TA muscle) was injected, corresponding to a total amount of 7.2 × 10^9^ particles. The same volume of Al-Rho suspension was injected in TA muscles. PBS-injected mice were used as controls. Tissues, including popliteal and inguinal DLNs, spleen, brain and blood, were collected at various time points post injection. Three mice were included per group at each time point for both injected materials and their controls. Other administration routes were compared to the standard i.m. injection, including s.c. injection of 20 μL FLBs in each hindlimb, and i.v. injection of 40 μL FLBs in the tail vein.

### Stereotactic cerebral injections

Mice were anaesthetized with ketamine and xylazine. Al-Rho suspension (0.5 μL) was stereotactically injected in the striatum using a 1 μL Hamilton syringe. Biodistribution of i.c. injected Al-Rho to cervical DLNs, assessed by serial sectioning of the whole cervical region, and spleen, was compared to biodistribution to the popliteal DLN and spleen of the same amount of Al-Rho injected in the TA muscle.

### Pharmacological and physical migration blockade

The prostaglandin analog BW245C, an agonist of the PGD2 receptor, was used to inhibit APC migration as previously reported [20]. Since BW245C is active for two days after injection, BW245C (100 nM, Cat.no.12050, Cayman Chemical, Ann Arbor, MI, USA) was injected twice in the TA muscle: it was first co-injected with FLBs at d0 and a second time alone at d2, and DLNs were removed for examination at d4. Untreated FLB-injected mice were used as controls. In another set of experiments DLNs were surgically ablated and mice immediately injected with FLBs in the TA muscle.

### Loss and gain of CCL2 function experiments

Exploratory analyses performed in MMF patients with ASIA [see Additional file 1: supplementary information section] yielded a CCL2 signal in the form of: (1) a selective increase of CCL2 in the serum of MMF patients compared to healthy controls; and (2) a given haplotype in the *CCL2* gene tending to be more frequent in MMF patients than in the general population. These results led us to use mouse models to explore the role of CCL2 in the biodisposition of particulate materials. Loss of CCL2 function studies were done using *CCL2*^−/−^ mice injected i.m. with 40 μL Al-Rho. Gain of CCL2 function experiments consisted first of i.m. co-injection of 10 μL murine rCCL2 (100 μg/ml; R&D, Minneapolis, MN, USA) with 40 μL Al-Rho. DLNs were removed at d4, spleen, brain and blood at d21. In other experiments murine rCCL2 was infused into the brain through a catheter stereotactically inserted into the striatum at d7 post-Al-Rho, fed by a subcutaneously implanted osmotic micropump fixed into the neck (0.25 μL/hour Alzet brain infusion kit, Charles River, L’Arbresle, France). rCCL2 was infused for 14 days (diffusion rate 180 pg/day), with or without rCCL2 i.m. injection concurrent with Al-Rho injection. At d21 post Al-Rho injection, animals were sacrificed, and blood and tissues were collected. For controls, osmotic pumps filled with PBS were used.

### Tissue preparation and particle counting

Mice under terminal anesthesia were transcardially perfused with PBS followed by ice-cold 4% paraformaldehyde (PFA) in 0.1 M phosphate buffer. Tissues and organs were removed, post-fixed in PFA for 4 hours at 4°C, immersed overnight at 4°C in a 30% sucrose solution, and quickly frozen. Whole brains were serially cut into coronal cryosections of 40 μm, spleen and muscle into 20 μm, and DLNs into 10 μm, and stored at −20°C until particle counting or treatment. Brain sections were successively deposited on 10 different Superfrost® slides in order to obtain 10 identical series, thus allowing determination of total particle content by multiplying by 10 the number of particles found in one series. A similar approach was used for DLNs and spleen. Blood was collected by heart puncture and 100 μL were smeared for particle counting.

### Immunohistochemistry and Morin staining

Immunostaining was done using commercial primary antibodies routinely used in the lab, raised against CD11b (1/200, AbD Serotec, Oxford, UK), F4/80 (1/50, AbCam, Cambridge, UK), GFAP (1/200, DakoCytomation, Trappes, France), vimentin (1/500 DakoCytomation), collagen IV (1/100 Millipore, Temecula, CA, USA), NG2 (1/200, Millipore, Molsheim, France), MAP2 (1/100, Sigma-Aldrich, Lyon, France), and IL1β (1/100, AbCam, Paris, France) or nonspecific mouse IgG (Jackson ImmunoResearch, Suffolk, UK). Then, biotinylated anti-rat and anti-rabbit antibodies (1/200, Vector Laboratories, Paris, France) were used accordingly and were revealed using Alexa fluor 488-conjugated streptavidin (1/200 Invitrogen, Cergy-Pontoise, France). Neuron labeling was done using NeuroTrace® blue fluorescent Nissl Stain according to the manufacturer” instructions (Invitrogen). Al was stained with Morin (M4008-2 G, Sigma-Aldrich) used as 0.2 g dissolved in a solution consisting of 0.5% acetic acid in 85% ethanol [21]. Formation of a fluorescent complex with Al was detected under a 420 nm excitation wavelength as an intense green fluorescence with a characteristic 520 nm emission. Notably, nanohybrids (Gd_2_O_3_) core encapsulated by polysiloxane shell were not positively stained by Morin. In contrast, when coated with Al(OH)_3_, these particles were strongly positive for Morin. Fluorescence microscopy and spectral analyses were done using Carl Zeiss light and confocal microscopes.

### Cell isolation from blood and tissues and flow cytometry

For blood cell immunophenotyping, 100 μL blood was treated with ethylenediaminetetraacetic acid (EDTA) and stained with fluorescein isothiocyanate (FITC)-conjugated antibodies. Erythrocytes were lysed using hypotonic lysis solution, and then cells were washed with (D)MEM and sorted using a MoFlo cell sorter (Beckman Coulter, Villepinte, France). Cells were extracted from tissues of exsanguinated mice perfused with PBS. Tissues were removed and freshly dissociated in (D)MEM. DLNs and spleen were dissociated in (D)MEM containing 0.2% collagenase-B (Roche Diagnostics, Meylan, France) and 0.2% trypsine-EDTA at 37°C for 45 minutes twice. Brain tissue was dissociated in 1% Trypsin-HBSS (Thermo Scientific HyClone, South Logan, UK) containing 100 U/mL DNase (Roche Diagnostics). Cell suspensions were filtered and counted. CD45^+^ or CD11b^+^ cells were isolated using magnetic cell sorting (MACS, Miltenyi Biotec, Paris, France) and stained with one of the following antibodies and their isotypes: FITC-conjugated anti-CD11b, FITC–conjugated anti–Ly-6C (GR1), FITC–conjugated anti-CD11c (BD-Pharmingen Bioscience, San Diego, CA, USA). Cells were sorted using a cell sorter. Populations presenting >90% purity were used. Sorted cells were cytospinned and stained with Hoechst-33342 for nucleus. Particle loaded cells were counted under a fluorescence microscope.

### Bone marrow transplantation experiments

GFP^+^ bone marrow (BM) cells were obtained by flushing the femurs of adult CAG-GFP mice and were injected retroorbitally (1 × 10^7^ cells per mouse) to four-week-old C57BL/6 mice, as previously described [15]. Recipient mice were irradiated at 9.0 Gy on d1 before transplantation, and were treated with 10 mg/kg/day ciprofloxacin for 10 days. Blood chimerism of >90% was controlled at three to four weeks post-transplantation.

### Statistical analyses

All experimental values are presented as means and standard deviation except when indicated. Statistical analyses used unpaired Student’s *t*-test (genotypes); *P* <0.05 was considered significant.

## Results

### Intramuscular alum-containing vaccine injection in mouse induces Al deposition in distant tissues

**Figure 1:**
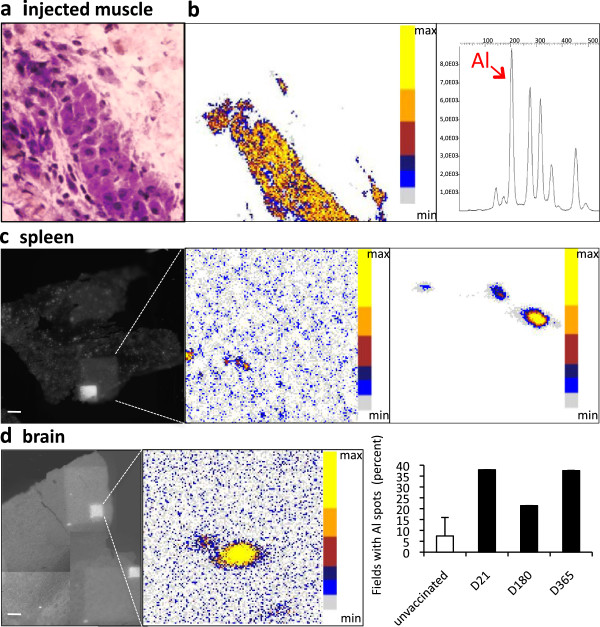
**Aluminum deposits in tissues following injection of alum-containing vaccine in the TA muscle. a)** Granuloma composed of PAS^+^ cells is formed in the injected muscle envelope; **b)** PIXE mapping shows muscle Al deposits in pseudocolors, with confirmatory Al emission spectrum (d21); **c)** Section of spleen tissue (left panel) displays the large 500 × 500 μm and restricted 100 × 100 μm protonized fields corresponding to the PIXE maps (middle and right panel, respectively) enclosing eligible Al spots (d21); **d)** Section of brain tissue (left left panel) displays the restricted 100 × 100 μm protonized field corresponding to the PIXE map (middle panel) enclosing eligible Al spot (d21); the number of fields containing one or more Al spots was increased at all tested time points compared to unvaccinated (right panel) mice. (bars: 100 μm). d, day; PIXE, particle induced X-ray emission, TA *tibialis anterior*.

Alum-containing vaccine (36 μL corresponding to 18 μg Al) was first injected in the TA muscles of C57Bl6 mice. It induced an acute inflammatory reaction which stabilized after d4 in the form of collections of typical alum-loaded MPs with large hematoxylin^+^ and Periodic Acid Schiff^+^ cytoplasm in muscle envelopes (Figure 1a). In parallel, the local Al tissue concentration determined by atomic absorption spectrometry decreased by 50% from injection to d4 and then remained stable until d21 (2,342, 1,122, and 1,180 μg/g of dry muscle tissue, respectively). Al was additionally located in muscle and distant tissues by PIXE [19]. Random scanning of 20 μm thick sections, sampled and processed with careful protection against environmental Al, disclosed significant Al signals in muscle, spleen and brain (Figure 1b-c). In brain, Al spots accounted for 38, 21, and 37% of 500 × 500 μm tested fields at d21, and months 6 and 12 (mo6 and mo12) post-injection, respectively (mean = 31.5%; n = 73 fields, Figure 1d). The dip at month 6, was either due to interindividual variations in aluminum handling or to sampling problems related to variable proportions of grey and white matter in the randomly scanned areas (see below). The spot size ranged from about 1 to 14 μm. By comparison, five unvaccinated mice showed only seven positive out of 94 tested fields (mean = 7.4%). These results confirmed that Al derived from alum can be translocated to, penetrate and persist in brain tissue [21-23]. Al depots detected in spleen and brain could have resulted from either physical translocation of alum particles, or *in situ* aggregation of soluble Al, or both.

### Fluorospheres injected into mouse muscle undergo lymphatic and systemic biodistribution

**Figure 2:**
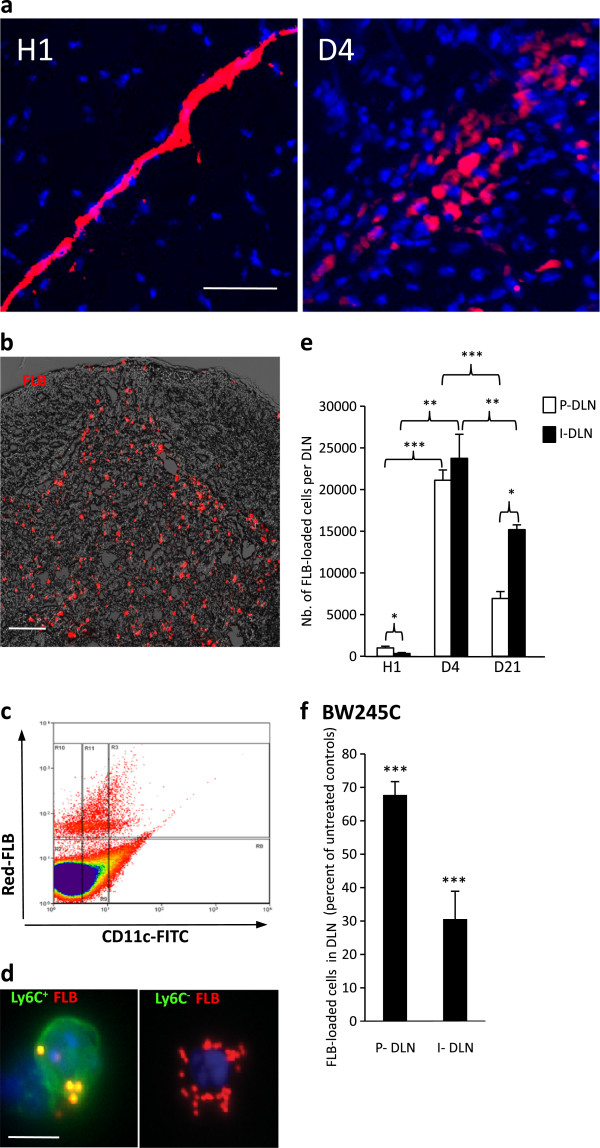
**FLB translocation in DLN following injection in TA muscle. a**) Marked translocation of FLBs in parafollicular areas of popliteal DLNs (d4); **b**) Flow cytometry showing that most FLB-loaded cells extracted from DLN express CD11c at either an intermediate or strong level (d4); **c)** Immunocytochemistry on CD11b^+^ cells extracted from DLNs were usually Gr1^+^/Ly6C^+^, especially when they had ingested a few particles (left), whereas heavily loaded ones were often Gr1^-^/Ly6C^-^ (right); **d)** The number of FLB-loaded cells peaked at d4 post-injection in both popliteal and inguinal DLNs; **e)** The migration inhibitor BW245C co-injected with FLBs in muscle markedly decreased the number of FLB-loaded cells detected in DLNs at d4 post-injection. The effect was more pronounced in the downstream inguinal DLN; **f)** The migration inhibitor BW245C co-injected with FLBs in muscle markedly decreased the number of FLB-loaded cells detected in DLNs at d4 post-injection. The effect was more pronounced in the downstream inguinal DLN; (histograms: n = 3 per group, mean + SD, * *P* <0.05, ** *P* <0.01, *** *P* <0.005; bars: 100 μm [a]; 5 μm [c]). d, day; DLNs, draining lymph nodes; FLBs, fluorescent latex beads; TA, *tibialis anterior*.

To examine if particles translocate to distant sites, we next injected polychromatic FLBs. A size of 500 nm was chosen as an approximation of the average size of alum agglomerates observed *in vivo*, allowing FLB visualization as individual spheres by confocal and fluorescence microscopes (resolution >200 nm). After i.m. injection of 20 μL suspensions, FLBs transiently peaked in free form in blood (1,200 + 400 FLBs per 100 μL) at hour 1. As early as 1 hour post-injection, some FLBs had also reached DLNs. I.m. injection of GFP^+^CD45^+^ cells, either pre-loaded with FLBs or coinjected with FLBs, showed no GFP^+^ cells translocation to DLNs at hour 1 (data not shown), indicating early cell-independent particle translocation to DLNs by lymphatic drainage of the muscle interstitial fluid [24]. In DLNs, however, most FLBs were cell-associated suggesting rapid capture by DLN resident cells. Within 24 hours, FLBs were phagocytosed by muscle CD11b^+^ MO/MPs. Phagocytes progressively cleared the particles away from the interstitium to form collections (Figure 2a), mainly located in muscle envelopes at d4.

**Figure 3:**
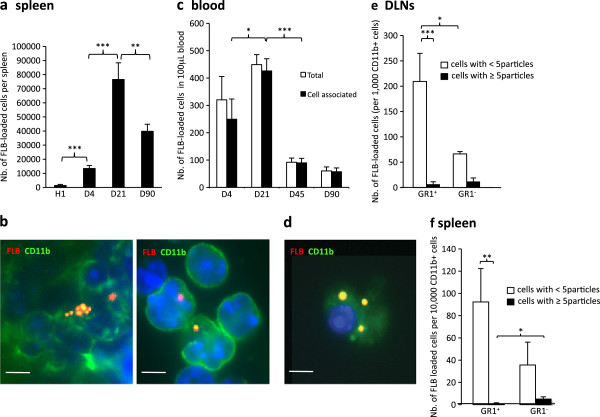
**FLB biodistribution in spleen and blood following injection in TA muscle. a)** The number of FLB-loaded cells peaked at d21 in spleen; **b)** In spleen, FLBs were detected in CD11b^+^ cells as assessed by immunohistochemistry (left) or after cell sorting (right); **c)** On blood smears, most FLBs were cell-associated from d4 and peaked at d21 post injection; circulating FLB-loaded cells were still detected at d90 endpoint; **d)** Circulating FLB-loaded cells were CD11b^+^ (d21); **e,f)** GR1/Ly6C immunophenotyping of CD11b^+^ cells that have ingested FLBs. Most are Gr1^+^/Ly6C^+^ both in DLNs at d4 (a) and in spleen at d21 (b). (histograms n = 3 per group, mean ± SD, * *P* <0.05, ** *P* <0.01, *** *P* <0.005; bars: 5 μm). d, day; FLB, fluorescent latex beads; n, number; TA, *tibialis anterior*.

At d4, FLBs had dramatically increased in DLNs, forming intracellular agglomerates in the interfollicular area (Figure 2b-e). Particle-loaded cells extracted from DLNs at d4 were CD45^+^, CD11b^+^, and more often GR1^+^/Ly6C^+^ (69% to 81%), and CD11c^+^, with either intermediate (46%) or high (22%) intensity (Figure 2a,c,d), thus corresponding to MO-derived inflammatory DCs and MPs [25]. Co-injection of FLBs with the synthetic prostaglandin analog BW245C, a compound known to inhibit DC migration [20], inhibited FLB translocation to DLNs at d4 by 32% in the popliteal and 69% in the inguinal DLNs, respectively (Figure 2f). This indicated prominent particle transport within phagocytic cells, at least downstream to popliteal DLN. At later time points, both the number of particle-loaded cells and the individual cell load markedly decreased in DLNs (Figure 2e). While decreasing in DLNs, FLBs dramatically increased in spleen from d4 to d21 (Figure 3a,b). As spleen is unplugged to lymphatic vessels, the particle transfer from DLNs to spleen implicated exit from the lymphatic system through the thoracic duct and circulation in the blood stream. Consistently, smears showed a similar d21 peak of FLB-loaded CD11b^+^cells in the circulation (Figure 3c,d). From d4, circulating FLBs were cell-associated (Figure 3d). Most FLB-loaded cells in blood, DLNs and spleen exhibited a few particles and were GR1^+^/Ly6C^+^ (Figure 3e,f). However, 22% to 33% were GR1^-^/Ly6C^-^ in spleen and had frequently incorporated >5FLBs, suggesting phagocytosis-associated maturation of inflammatory MO-derived cells [20,25,26]. FLB-loaded cells had markedly decreased in spleen at d90. Although declining after d21, FLB-loaded cells were still detected in blood at d45 and d90.

### Fluorosphere incorporation into brain is delayed and depends on prior cell loading in peripheral and lymphoid tissues

**Figure 4:**
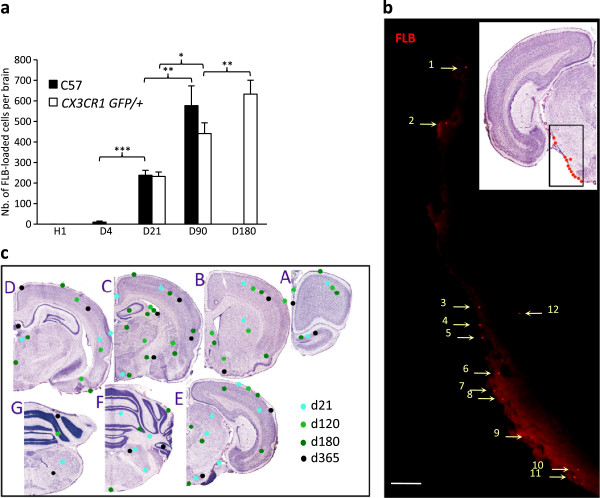
**Brain translocation of FLBs following injection in TA. a)** Cerebral translocation of FLBs was delayed but relentless until the d90 endpoint in C57 mice and the d180 endpoint in the *CX3CR1*^GFP/+^ mouse; **b)** Unstained section of the brainstem in a C57 mouse at d21 post-injection showing FLBs mostly distributed in the subpial region; **c)** FLBs distribution in the brain: areas enriched in FLBs were reported on semi-serial rostro-caudal sections of mouse brain stained by Cresyl violet (A to G), using dots of different colors according to the considered time point (d21 to d365) after i.m. injection. Report was done regardless of the number of enclosed particles in each selected area. Note that FLBs were always predominantly found in the grey matter without prominent accumulations at any specific neuroanatomical site. (histograms: n = 3 per group, mean + SD, * *P* <0.05, ** *P* <0.01, *** *P* <0.005; bar in b: 50 μm). d, day; FLBs, fluorescent latex beads; n, number; TA, *tibialis anterior*.

**Figure 5:**
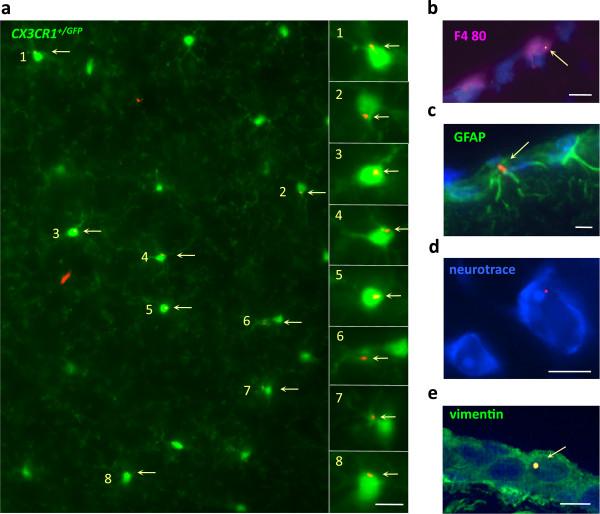
**FLBs in various neural cells. a)** Unstained section of the brain parenchyma of a *CX3CR1*^GFP/+^ mouse at d90 post-injection showing individual FLBs in a significant proportion of GFP^+^ ramified microglial cells; **b-e)** In brain of C57 mice at d21post-injection, FLBs were detected in F4/80^+^ perivascular macrophages (b), GFAP^+^ astrocytes (c), neurotrace®^+^ neurons (d), and vimentin^+^ pial cells (e); (bars: 10 μm). d, day; FLB, fluorescent latex beads.

Particles were detected in brain mainly from d21 post-injection. After d21 post-i.m. injection, FLBs gradually increased in brain until the d90 endpoint in the C57Bl6 mouse (Figure 4a,b) and until the d180 endpoint in the *CX3CR1*^GFP/+^ mouse conventionally used to study resident microglia (Figures 4a and 5a). FLBs were predominantly found in the grey matter (82% to 95%), regardless of the amount of injected FLBs (4, 10, 20 μL), vaccine co-injection (36 μL), or post-injection time from d21 to d365. Some FLBs were detected in leptomeninges (9%) and in the white matter (9%) at d21, but these locations became rare at later time points. FLBs were <5% in choroid plexus (Table 1). Comparative FLB distribution at month 3, month 6 and month 12 showed no prominent accumulations of particles at any neuroanatomical location (Figure 4c). FLBs were usually detected in brain as single particles located within or at the surface of cells; 37% to 62% of particles could be reliably assigned to a given cell subset by immunohistochemical screening. At d21, particles were mainly associated with perivascular CD11b^+^ MPs, but at d90 they were also found in deep ramified CX3CR1^+^microglia (Figure 5a). Particles were also detected in GFAP^+^ astrocytes, MAP2^+^ or Neurotrace-stained neurons, and vimentin^+^ leptomeningeal cells (Figure 5b-e), and in NG2^+^ oligodendroglial progenitors/pericytes (not shown). FLB incorporation into GFP^+^ resident ramified microglia of *CX3CR1*^GFP/+^ mice increased by up to 26-fold the d21 value at d180.

**Table 1:** Distribution of particles (percent of total) according to post-injection time

**Time post-injection** **Localization**	**D21**	**D90**	**D180**	**D365**
Choroid plexus	0%	5%	5%	3%
Leptopmeninges	9%	5%	0%	3%
Parenchyma	91%	90%	95%	94%

**Figure 6:**
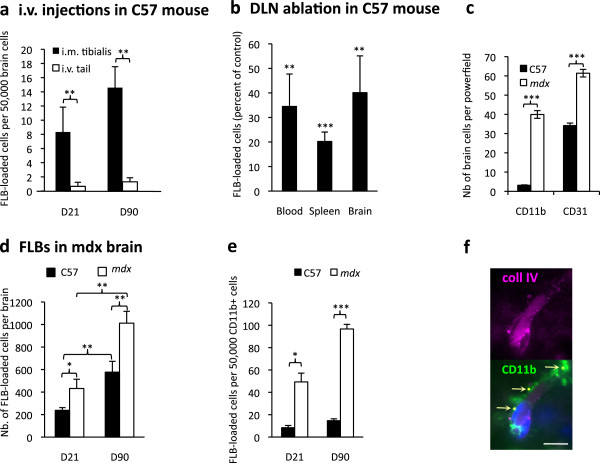
**Mechanisms of FLBs translocation. a)** Compared to the i.m. route, direct injection of FLBs in the tail vein of C57 mice was associated with almost no brain translocation at both d21 and d90 post-injection; **b)** Popliteal and inguinal DLN ablation was associated with a marked decrease of FLB-loaded cells in blood, spleen and brain at d21 post-injection; **c)** The *mdx* mouse with altered BBB showed a marked increase of the perivascular CD11b^+^ cell population, and significant angiogenesis assessed by an increase of CD31^+^ endothelial cells, compared to normal C57 mice; **d**-**e)***Mdx* mice showed increased incorporation of FLBs in brain; compared to C57 mice, *mdx* mice had increased FLB neurodelivery at both d21 and d90, as assessed by both histology (d) or after CD11b^+^ cell sorting (e); **f)** At d21, FLBs were mainly detected outside capillary basement membranes immunostained for collagenIV (upper panel), closely associated with CD11b^+^ perivascular macrophages (lower panel); (histograms: n = 3 per group, mean + SD, * *P* <0.05, ** *P* <0.01, *** *P* <0.005; bar in d: 10 μm). d, day; DLN, draining lymph nodes; FLB, fluorescent latex beads; n, number.

**Figure 7:**
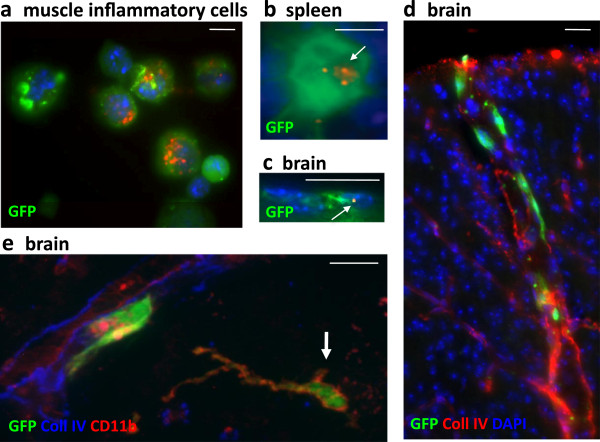
**GFP**^**+**^**BM chimeric mice. a-c)** Chimeric mice injected intramuscularly with FLBs showed GFP^+^ BM-derived cells enclosing FLBs among inflammatory cells extracted from the injected muscle (a) at d4 after FLB injection, in spleen (b) and brain (c) at d33 after FLB injection. **d-e)** Chimeric mice showed incorporation of GFP^+^ cells in the brain, mainly in the form of perivascular cells in the cortex (d) and, occasionally, in more deeply located ramified CD11b^+^ cells (e, arrow) at d180 post-BM transplantation. (bars: 10 μm). BM, bone marrow; d, day; FLB, fluorescent latex beads.

Importantly, compared to i.m. injection, the same FLB amount injected in the tail vein resulted in virtually no cerebral entry at d21 and d90 in C57Bl6 mice (Figure 6a). Moreover, ablation of popliteal and inguinal DLNs before FLB injection in TA muscle resulted in 60% to 80% reduction of FLB incorporation into blood, spleen and brain compartments at d21 (Figure 6b). Thus, cell uptake in muscle and DLNs, and subsequent cell traffic to blood crucially contributed to delayed particle translocation to spleen and brain (Figure 6a-f). Consistently, by injecting FLBs into the muscle of GFP^+^BM chimeric mice obtained by transplanting GFP^+^BM-derived cells to irradiated syngenic C56*Bl6* mice [15], we detected FLB-loaded GFP^+^cells in these organs (Figure 7a,b,c) and observed delayed incorporation of donor-derived cells in brain (Figure 7d,e).

This BM transplantation model is known to be associated with irradiation-induced BBB alteration. Dystrophin-deficient *mdx* mice also have chronically altered BBB [27]. As a corollary, compared to age-matched controls, they show significantly more CD31^+^ brain capillaries, and a dramatic increase of perivascular CD11b^+^ macrophages (Figure 6c) at the expense of deep ramified microglia. FLB injection in *mdx* mouse muscle resulted in increased brain incorporation of particles at both d21 and d90, as assessed by both histology and cytospins of CD45^+^/CD11b^+^cells extracted from brain (Figure 6d,e,f). Thus, BBB alteration and/or the associated inflammatory/angiogenic response likely favors brain incorporation of circulating particle-loaded cells.

### Fluorescent nanohybrids coated with Al(OH)_3_ undergo CCL2-dependent systemic scattering and brain penetration

**Figure 8:**
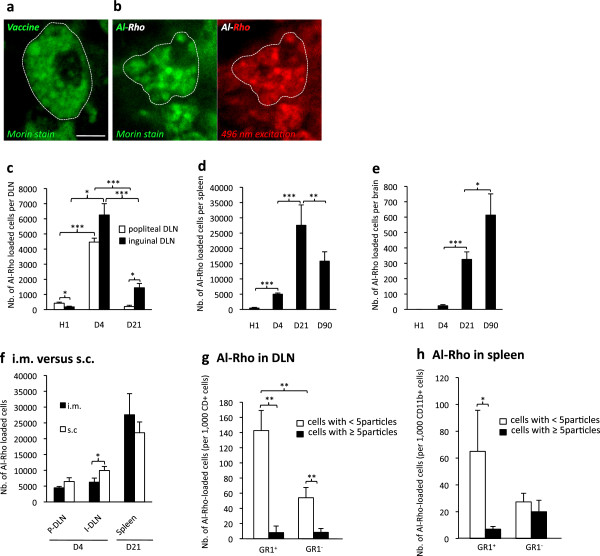
**Biodistribution of Al particles. a)** Morin stain for aluminum shows rounded alum cytoplasmic agglomerates within muscle macrophages after i.m. vaccine administration in C57 mouse; **b)** Morin stain confirms that phagocytosed Al-Rho nanohybrids are associated with Al and form particles similar in size to alum agglomerates; **c-e)** Al-Rho nanohybrids show a time-dependent distribution in DLNs, spleen, and brain strikingly similar to that of FLBs; **f)** Al-Rho injected by the s.c. route translocate to DLNs and spleen, as observed with the i.m. route; **g,h)** Ly6C immunophenotyping of CD11b^+^ cells that have ingested Al-Rho: most are Gr1^+^/Ly6C^+^ both in DLNs at d4 (**g**) and spleen at d21 (**h**). (histograms: n = 3 per group, mean ± SD, * *P* <0.05, ** *P* <0.01, *** *P* <0.005; bar in a: 10 μm). d, day; DLN, draining lymph nodes; FLBs, fluorescent latex beads; n, number.

For confirmatory experiments we constructed fluorescent particles mimicking alum. Rhodamine nanohybrids [28] were covalently coated with a Al(OH)_3_ shell. As assessed by the Morin stain for alumimum, these Al-Rho particles were avidly phagocytosed after i.m. injection and formed intracellular agglomerates similar in size to the vaccine adjuvant (Figure 8a,b). Biodistribution of the alum fluorescent surrogate injected into TA muscle was strikingly similar to that of FLB (Table 2), including d4 peak in DLNs, d21 peak in spleen, delayed entry in brain, and main association with GR1^+^/Ly6C^+^ MOs in tissues (Figure 8c-h). Compared to i.m. injection, s.c. injection of Al-Rho particles was associated with an even higher rate of diffusion to DLNs (Figure 8f), a finding consistent with the presence of abundant migratory DCs in skin.

**Table 2:** Time of peak observation and peak value of particle loaded cells in studied organs (total number ± SD)

**Particle**	**Popliteal DLN**		**Inguinal DLN**		**Spleen**		**Blood**		**Brain**	
	**Peak**	**Number of loaded cells**	**Peak**	**Number of loaded cells**	**Peak**	**Number of loaded cells**	**Peak**	**Number of loaded cells**	**Endpoint**	**Number of loaded cells**
**FLB**	D4	21,117 ±1,235	D4	23,746±2,880	D21	76,503±11,850	D21	9,878±792	D90	577±96
**Al-Rho**	D4	4,462 ± 257	D4	6,253±745	D21	27,570±6,670	D21	7,546±1,034	D90	613±137

**Figure 9:**
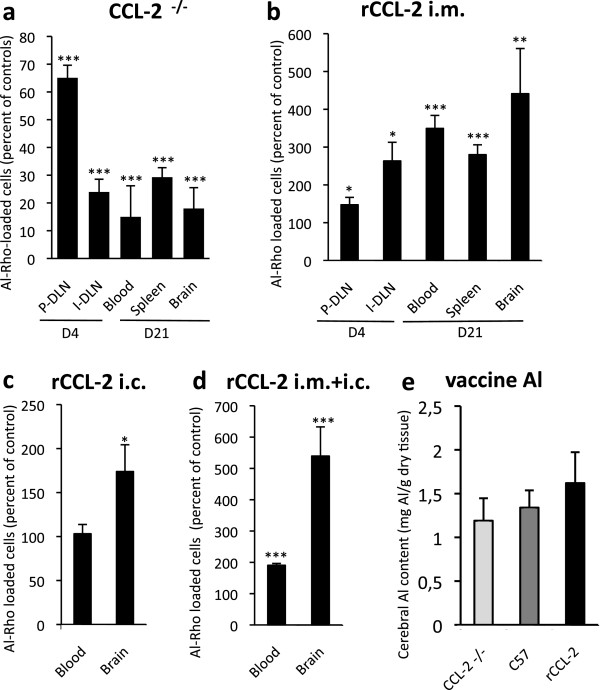
**CCL2-dependent systemic translocation of Al-particles. a**) CCL2 deficient mice show a dramatic decrease of Al-Rho translocation from the injected muscle to inguinal DLN, blood, spleen and brain, as compared to their respective controls (100%). Note that the difference is significant but less pronounced for popliteal DLN; **b)** rCCL-2 co-injection with Al-Rho is associated with a marked increase of Al-Rho translocation from the injected muscle to inguinal DLN, blood, spleen and brain, compared to their respective controls (100%). Note that the difference is significant but less pronounced for popliteal DLN; **c)** rCCL-2 infused by an osmotic micropump into the striatum for 15 days is associated with a significant increase of Al-Rho translocation from the injected muscle to brain; **d)** Combined i.m. and i.c. injection of rCCL2 is associated with a dramatic increase of FLB translocation from muscle to both blood and brain; **e)** Alum-containing vaccine injected into muscle of CCL-2-deficient, normal, and rCCL-2 mice was associated with a trend of CCL-2-dependent increase of Al concentration levels in brain; (histograms: n = 3 per group, mean ± SD, * *P* <0.05, ** *P* <0.01, *** *P* <0.005, except [e]: n = 10 per group, mean ± SEM). Al-Rho, Al(OH)_3_ rhodamine nanohybrid; DLN, draining lymph nodes; FLB, fluorescent latex beads; n, number; SEM, standard error of the mean.

On the grounds of a human SNP study, we performed CCL2 gain and loss of function experiments to investigate the role of CCL2-responsive cells in particle scattering and neurodelivery. Injection of Al-Rho particles into the TA muscle of CCL2-deficient mice decreased particle incorporation by 35% into popliteal DLN and by 76% in inguinal DLN at d4, and by 71%, 85% and 82% in spleen, blood, and brain, respectively, at d21 (Figure 9a). Conversely, Al-Rho particle biodistribution increased in different gain of CCL2 function experiments (Figure 9b-d). I.m. co-injection of Al-Rho with murine recombinant CCL2 (rCCL2: 1 μg) increased particle incorporation by 47% into popliteal and 163% into inguinal DLN (d4), and by 180% in spleen, 274% in blood, and 341% in brain (d21).

**Figure 10:**
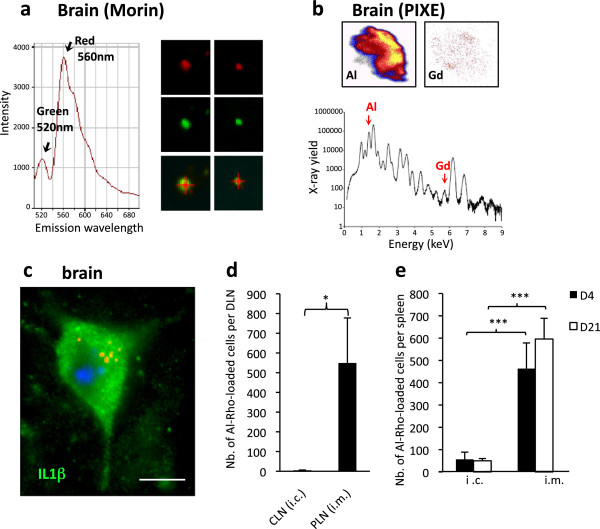
**Al-Rho particles remain in brain and may induce inflammation. a)** Al-Rho nanomaterial detected in brain by rhodamine fluorescence (upper row and emission spectrum at 560 nm) remains associated with Al as assessed by Morin stain (middle row and emission spectrum at 520 nm); **b)** Al-Rho nanomaterial detected in the brain by PIXE. Al coating colocalizing with Gd core assesses the integrity of Al-Rho nanohybrid after translocation; **c)** In mice with i.m. co-injection of Al-Rho and rCCL-2, particle incorporation into neural cells was associated with immunohistochemical expression of IL1beta; **d)** Stereotactic injection of Al-Rho into the striatum was associated with no translocation to cervical LNs (CLN) at d4, contrasting with conspicuous translocation to popliteal LNs (PLN) observed when the same particle amount was injected in TA muscle; **e)** Stereotactic injection of Al-Rho into the striatum, compared to similar injection into muscle, was associated with very little translocation to spleen at both d4 and d21. (histograms: n = 3 per group, mean ± SD, * *P* <0.05, ** *P* <0.01, *** *P* <0.005; bar in c: 10 μm). Al(OH)_3_ rhodamine nanohybrid; d, day; LN, lymph nodes; n, number; PIXE, particle induced X-ray emission;TA, *tibialis anterior*.

Morever, slow intracerebral (i.c.) infusion of CCL2 by an osmotic pump (180 pg/day during 15 days starting at d7 after Al-Rho i.m. injection) increased particle incorporation into brain by 74% at d21 compared to PBS control. The combination of i.m. injection and i.c. infusion of rCCL2 increased particle incorporation into the brain by 539%. Despite important interindividual variations, a consistent trend of CCL2-dependent increase of Al brain levels was detected 21 days after i.m. injection of 40 μL of alum-containing vaccine (Figure 9e). Taken together these results indicate that after i.m. injection, particles associated with inflammatory MOs can enter the brain using a CCL2-dependent mechanism, possibly through a Trojan horse mechanism. Importantly, Al-Rho particles gaining access to the brain after i.m. injection remained intact since they were still coated with Al(OH)_3_ as assessed by both Morin stain (Figure 10a), and PIXE (Figure 10b). Their incorporation in neural cells was consistently associated with the expression of IL-1β (Figure 10c), a reliable marker of particle-induced NALP3 inflammasome activation [29].

### Fluorescent nanohybrids coated with Al(OH)_3_ are retained in brain

An apparently irreversible accumulation of nanomaterials after i.m. injection was unique to brain tissue which lacks conventional lymphatic pathways and may retain immune cells [30]. We stereotactically injected 0.5 μL Al-Rho in the striatum of C57*Bl6* mice, and counted particles in cervical LNs, blood, and spleen at d4 and d21. Compared to the same amount of Al-Rho injected in the TA muscle, i.c. injection was associated with almost no particle translocation to regional DLNs (Figure 10d), and the appearance of eight-fold fewer particles in spleen (Figure 10e). Since 25 free Al-Rho particles per 100 μL were detected in blood at hour 1, it is likely that the rare particles subsequently detected in spleen reflected direct particle passage into blood during i.c. injection. It seems, therefore, that lack of recirculation likely contributed to progressive cerebral particle accumulation.

## Discussion

Particles injected by the i.m. or s.c. route gained access to distant tissues. Latex and Al-Rho particles showed closely similar biodistribution, suggesting a shared basic scattering mechanism. Initial cell uptake in peripheral and DLN tissues and subsequent transport within inflammatory MO-derived cells was critically involved, as indicated by immunophenotyping, cell migration blockade and DLN ablation. Cells were heavily loaded with particles soon after i.m. injection but usually contained only one to two particles after d4 and downstream the popliteal DLN, pointing to either dilution by cell division [31] or particle dispatching to other cells [32] within DLNs. Previous studies have reported particle cell transport from skin to DLNs [25] but downstream particle fate remained largely unexplored [33]. There is strong evidence that, in inflammatory conditions, all DCs reaching DLNs do not die locally but may rather gain access to the blood through efferent lymphatics and the thoracic duct, and present antigens in spleen and bone marrow [33]. Ingested adjuvant particles boost this phenomenon which in turn likely favors their translocation from the injection point to distant sites as: (i) alum induces rapid differentiation of monocyte-lineage cells into APCs [34] and stimulates their migration to DLNs [35],(ii) beryllium hydroxide, a closely similar particulate adjuvant, strongly stimulates DC egress through efferent lymphatics [36]; and, as shown herein, (iii) Al deposits may be detected by PIXE in spleen and brain after i.m. injection of alum.

Delayed and slowly progressive particle accumulation occurred in intact brains. Experiments using the parabiosis model [37] or avoiding brain irradiation prior to BM transplantation [38] have shown that endogenous microglia are not replenished by the periphery under normal central nervous system (CNS) conditions. Although low chimerism inherent in these experimental approaches may lead to some underestimation of slow microglia turnover from the periphery [39], a more likely explanation of our findings is that particles exert stimulatory effects on myeloid cell trafficking [36]. Both latex particles and aluminum hydroxide agglomerates promote inflammation [40,41] and non-specific immune stimulation can increase monocyte transendothelial migration by up to 20-fold in *in vitro* models of the BBB [42]. Consistently, i.m. injection of rCCL2 strongly increased particle incorporation into intact brain while CCL2-deficient mice had decreased neurodelivery. rCCL2 likely induced the exit of inflammatory MOs and hematopoietic stem and progenitor cells from BM [43], followed by their transmigration to the injected muscle and to DLNs [44], prior to particle loading and dissemination. Cerebral infusion of low doses of rCCL2, mimicking pathological states attracting inflammatory monocytes, also increased particle neurodelivery. Intracerebral particles translocated with time from perivascular macrophages to the sentinel network of parenchymal microglia and to other resident neural cells and likely failed to recirculate, thus explaining their progressive cerebral accumulation.

## Conclusions

Taken together, our results indicate that, similarly to intracellular bacteria [45], nanomaterials can be transported by MO-lineage cells to DLNs, blood and spleen, and, similarly to HIV [46] and other pathogens [47], may use CCL2-dependent MO transmigration across the BBB to enter the brain. This occurs at an extremely low rate in normal mice, the percentage of injected particles found in tissues being estimated at 1:10^5^ in d21 spleen and 1:10^7^ in d90 brain, consistent with the excellent tolerance of almost all individuals to limited doses of alum and other injected particles. Neurodelivery of nanomaterials significantly increased in mice with either a weak BBB or high tissue levels of CCL2, as previously suspected for pathogens in humans [48]. On the one hand, such a cerebral incorporation of nanomaterials injected into tissues should be regarded as an interesting characteristic in the setting of therapeutic strategies targeting the CNS. On the other hand, alum has high neurotoxic potential [49], and planning administration of continuously escalating doses of this poorly biodegradable adjuvant in the population should be carefully evaluated by regulatory agencies since the compound may be insidiously unsafe. It is likely that good tolerance to alum may be challenged by a variety of factors including overimmunization, BBB immaturity, individual susceptibility factors, and aging that may be associated with both subtle BBB alterations and a progressive increase of CCL2 production [50].

## Abbreviations

Al-Rho: Al(OH)_3_ rhodamine nanohybrid; APC: antigen presenting cells; ASIA: autoimmune/inflammatory syndrome induced by adjuvant; BBB: blood brain barrier; BM: bone marrow; CCL2: chemokine (C-C motif) ligand 2; CNS: central nervous system; d: day; DC: dendritic cells; DEG: diethylene glycol; DLNS: draining lymph nodes; (D)MEM: (Dulbecco’s) modified Eagle’s medium; DMSO: dimethyl sulfoxide; EDTA: ethylenediaminetetraacetic acid; FITC: fluorescein isothiocyanate; FLB: fluorescent latex bead; GFAAS: graphite furnace atomic absorption spectrometry; IL: interleukin; mdx: dystrophin deficient mouse; MCP1: monocyte chemoattractive protein 1; MMF: macrophagic myofasciitis; MO: monocyte; mo: month; MP: macrophage; PBS: phosphate-buffered saline; PFA: paraformaldehyde; PIXE: proton induced X-ray emission; SNP: single nucleotide polymorphism; TA: *tibialis anterior* muscle; THGA: transversely heated graphite atomizer.

## Competing interests

The authors declare they have no competing interests.

## Authors’ contributions

ZK carried out animal experiments and tissue processing and participated in data analysis; CC carried out molecular genetics studies; FJA contributed clinical data; VI processed tissue for PIXE and participated in their analysis; FL participated in surrogate particles production; CE carried out Al determination in tissues; MMY and PM participated in the PIXE analysis, XD carried out confocal analysis; OT conceived and contributed surrogate particles; RKG conceived and coordinated the study, analyzed data and drafted the manuscript; JC designed the study, performed animal experiments, analyzed data and prepared the figures, and participated in the manuscript writing. All authors read and approved the final manuscript.

## Pre-publication history

The pre-publication history for this paper can be accessed here:

http://www.biomedcentral.com/1741-7015/11/99/prepub

## Supplementary Material

Additional file 1 **Appendix.** Supplementary material. Click here for file
