# Supplementary material for: Slow CCL2-dependent translocation of biopersistent particles from muscle to brain
Source: BMC Med. 2013 Apr 4;11:99. doi: 10.1186/1741-7015-11-99 (PMC3616851; doi:10.1186/1741-7015-11-99)
Supplement: Additional file 1 — Appendix. Supplementary material. [file 1741-7015-11-99-S1.pdf]

Appendix. Supplementary material

Relevant analyses in patients with MMF-associated ASIA.

Detection of single nucleotide polymorphisms (SNPs) in chemokine genes was previously used to identify key molecular pathways involved in the pathophysiology of complex diseases involving MO lineage cells (Combadière 2007). In this section we looked for possible signals linked to chemokines attracting inflammatory monocytes in patients with post-vaccinal macrophagic myofasciitis syndrome. A preliminary serum cytokine screen draw attention to the CCL2/MCP-1 chemokine (Cadusseau et al., in preparation). In this study, simultaneous quantitation of 30 cytokines, including 10 chemokines, was performed in serum of 44 patients with MMF (M/F sex ratio 16/28, mean age range 25-70, mean 46.1yrs), 44 age and sex matched healthy controls (sex ratio 16/28, age range 39-60, mean 48.5yrs), and 10 unmatched inflammatory controls with various inflammatory neuromuscular disorders, using the Luminex^{R} technology, with the Bio-Plex Pro Human Cytokine 27-plex Assay M50-0KCAF0Y from Bio-Rad (Hercules, CA) enriched with simplex sets for CCL7 (MCP-3) and CXCL1 (KC/Gro-alpha), and the quantikine DCX-310 ELISA kit for CX3CL1 (Fractalkine) purchased from R&D systems Europe (Lille, France). As assessed by the Mann-Whitney *U*-test, MMF patients showed significantly increased mean CCL2/MCP-1 levels compared to healthy subjects ($p<0.0001$; supplementary Fig. 1). None of the other tested molecules (including chemokines CCL3, CCL4, CCL5, CCL7, CCL11, CXCL8, CXCL1, CXCL10, and CX3CL1, p= NS) were increased. Inflammatory controls showed no increase of serum CCL2/MCP-1 compared with both MMF and healthy subjects (p=NS). Next, *CCL2* polymorphisms were determined in a larger series of MMF patients including those screened for serum chemokines.

S1 Patients and Methods

S1.1 Patients.

CCL2 polymorphisms were determined in a monocentric retrospective series of 252 patients with MMF syndrome and were correlated with 516 controls using a previously described procedure [Suppl 1,2]. Both patients and healthy controls were adult caucasians. Inclusion criteria were as follows: (i) onset of clinical manifestations posterior to alum-containing vaccine administration; (ii) diffuse arthro-myalgias lasting >6 months and/or profound fatigue lasting >6 months and/or disabling cognitive deficiency affecting attention and memory; (iii) histologic MMF assessed by deltoid muscle biopsy by one of the myopathologic centers that described the entity (i.e. Créteil, Paris, Marseille and Bordeaux, France), with time elapsed from last vaccine shot to diagnostic muscle biopsy >18 months; (iv) no other disease that could explain the manifestations. Most

were females (69%) and were at the middle age at time of biopsy (median 45 years, range 17-74). They had received 1 to 17 i.m. alum-containing vaccine administrations (mean 5.2) in the 10 years before MMF detection. They complained of diffuse arthro-myalgias (86%), disabling chronic fatigue (78%), and overt cognitive alterations (50%). The median delay from the last vaccine administration to biopsy was 66 months (range 18-219).

Thus 3 major criteria proposed for ASIA- autoimmune/inflammatory syndrome induced by adjuvants were met [supplementary reference 3], including (1) "exposure to adjuvant prior to clinical manifestations" (i.e. aluminium oxyhydroxide); (2) "the appearance of typical clinical manifestations" including: myalgia; arthralgias; chronic disabling fatigue; unrefreshing sleep or sleep disturbances; cognitive impairment and memory loss; and (3) "typical biopsy of involved organs", i.e. MMF. According to the principles expressed in the Helsinki Declaration, all patients gave written individual informed consent to participate to the clinico-genetic study and approval was obtained from our institutional review board (registration number 2010-A01338-31 at AFSSAPS, and 11-042 at CPP-Ile-de-France IX).

S1.2 Genotyping.

Genomic DNA was extracted after patient informed consent from saliva using OrageneTM technology or from frozen muscle biopsy samples used for diagnosis stocked by the biobank of Créteil. Polymorphisms in the *CCL2* gene were identified with minor groove binder PCR-based amplification technology from Applied Biosystems (Applied Biosystems Division, Cheshire, UK), including SNPs which positions relative to the adenine in ATG are -*3938* [rs1860189], -*2578* [rs1024611], and -*927* [rs3760396], -289 [rs2857656], +837 [rs2857657]), and +1616 [rs 1390000]. Real-time detection and analyses were performed on an ABI 7700 thermocycler (Applied Biosystems). All genotypes were tested for Hardy–Weinberg equilibrium. All comparisons between cases and controls used the standard *Chi*2 test with 95% CIs (GraphPad Prism 4.0).

S2 Results

We first evaluated the distribution of 2 previously reported SNPs of *CCL2/MCP-1* (-2578A>G [rs1024611], -927G>C [rs3760396]) associated with chemokine gain of function [supplementary references 1,4,5]. The two SNPs were in complete linkage disequilibrium (D'=-1; r2=0.08) and generated three haplotypes (supplementary table 1). The haplotype frequencies distribution derived from the two SNPs did not significantly differ between cases and controls ($\chi 2=3.95$ with 2df, $p=0.139$). Nevertheless, the -927G allele was carried out by only one haplotype, AG, that tended to be more frequent in cases (0.204 vs 0.163) while the two other haplotypes

carrying the -927C allele were less frequent in cases than in controls. Compared to the most frequent AC haplotype, the AG haplotype was associated with a slightly increased risk for disease (OR=1.28 [0.96-1.70], p=0.088) (supplementary table 1), a result consistent with what was observed in single locus analysis (data not shown). In a further haplotype analysis including additionnal SNPs in the *CCL2/MCP-1* gene (-3938 [rs1860189] and +837[rs2857657]), the -927G allele was still carried out by only one haplotype that was more frequent in cases than in controls (data not shown). Thus, despite the small number of tested samples inherent to rare conditions, a variation could be detected in the CCL2/*MCP-1* gene in alum-intolerant patients.

All MMF patients used for serum analysis were genotyped: 43 out of 44 had at least one polymorphism detected in the *CCL2/MCP-1* gene, and 20 had two of them in various combinations (-3938 [n=13], -2828 [n=25], -927 [n=14], +837 [n=14]). No correlation could be established between CCL2/MCP-1 serum levels and a given genotype (data not shown). This could be explained by (i) tissue rather than vascular/blood cell production of CCL2/MCP-1; (ii) unappreciated temporal variations in the chemokine release; or (iii) possible interference with other factors increasing CCL2/MCP-1 production, including other endogenous traits, ageing or unknown exogenous stimuli. Notably, intramuscular alum injection by itself does not induce detectable cytokine changes in serum [supplementary reference 6]. In contrast it upregulates a set of genes such as *CCR2* and other chemokine receptors [supplementary reference 6], which may favour cell migration. Although a lot remains to be understood in the pathophysiology of ASIA [supplementary reference 7,8], our patients data incited us to investigate the role of phagocytic cells and of the CCL2:CCR2 signaling in the biodisposition of nanomaterials in mice.

Supplementary references

[1] Combadière C, Feumi C, Raoul W, Keller N, Rodéro M, Pézard A, Lavalette S, Houssier M, Jonet L, Picard E, Debré P, Sirinyan M, Deterre P, Ferroukhi T, Cohen SY, Chauvaud D, Jeanny JC, Chemtob S, Behar-Cohen F, Sennlaub F. CX3CR1-dependent subretinal microglia cell accumulation is associated with cardinal features of age-related macular degeneration. J Clin Invest 2007;117:2920-2928.

[2] Rodero M, Marie Y, Coudert M, Blondet E, Mokhtari K, Rousseau A, Raoul W, Carpentier C, Sennlaub F, Deterre P, Delattre JY, Debré P, Sanson M, Combadière C. Polymorphism in the microglial cell-mobilizing CX3CR1 gene is associated with survival in patients with glioblastoma. J Clin Oncol 2008;26:5957-5964.

[3] Shoenfeld, Y.; Agmon-Levin, N. ASIA- autoimmune/inflammatory syndrome induced by adjuvants. J. Autoimmun. 2011, 36, 4-8.

[4] Flores-Villanueva PO, Ruiz-Morales JA, Song CH, Flores LM, Jo EK, Montaño M, Barnes PF, Selman M, Granados J. A functional promoter polymorphism in monocyte chemoattractant protein-1 is associated with increased susceptibility to pulmonary tuberculosis. J. Exp. Med., 2005, 19, 202(12):1649-58.
[5] Nyquist P, Zhang J, De Graba TJ. The -928 G/C and -362 G/C single nucleotide polymorphisms in the promoter of MCP-1: Increased transcriptional activity and novel binding sites. Cerebrocasc Dis 2010;29:242-247.
[6] Mosca F, Tritto E, Muzzi A, Monaci E, Bagnoli F, Iavarone C, O'Hagan D, Rappuoli R, De Gregorio E. Molecular and cellular signatures of human vaccine adjuvants. Proc Natl Acad Sci U S A. 2008;105:10501-6.
[7] Katzav A, Kivity S, Blank M, Shoenfeld Y, Chapman J. Adjuvant immunization induces high levels of pathogenic antiphospholipid antibodies in genetically prone mice: another facet of the ASIA syndrome. Lupus. 2012;21:210-6.
[8] Perricone C, Agmon-Levin N, Valesini G, Shoenfeld Y. Vaccination in patients with chronic or autoimmune rheumatic diseases: the ego, the id and the superego. Joint Bone Spine. 2012;79:1-3.

Supplementay figure 1: Serum CCL-2 levels in MMF patients compared to healthy subjects and inflammatory controls.

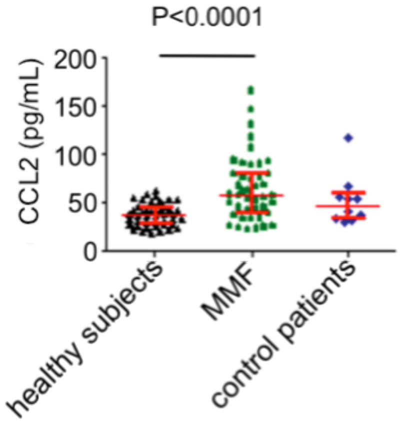

Supplementary table 1: Frequencies of haplotypes generated by SNPs in the *CCL2/MCP-1* gene of patients with alum intolerance compared to controls.

Polymorphisms		Haplotype Frequencies		Haplotypic Odds Ratio [95%CL]
CCL2_2578	*CCL2_927*	Controls (N=516)	Cases (N=252)	
G	C	0.271	0.245	0.936 [0.729 - 1.201] p=0.602
A	C	0.566	0.555	reference
A	G	0.163	0.204	1.280 [0.964 - 1.700] p=0.088
